# Tuning the reactivity of nitriles using Cu(ii) catalysis – potentially prebiotic activation of nucleotides†
†Electronic supplementary information (ESI) available. See DOI: 10.1039/c8sc02513d


**DOI:** 10.1039/c8sc02513d

**Published:** 2018-07-25

**Authors:** Ziwei Liu, Angelica Mariani, Longfei Wu, Dougal Ritson, Andrea Folli, Damien Murphy, John Sutherland

**Affiliations:** a MRC Laboratory of Molecular Biology , Francis Crick Avenue, Cambridge Biomedical Campus , CB2 0QH , UK . Email: johns@mrc-lmb.cam.ac.uk; b School of Chemistry , Cardiff University , Park Place , Cardiff CF10 3AT , UK

## Abstract

During the transition from prebiotic chemistry to biology, a period of solution-phase, non-enzymatic activation of (oligo)nucleotides must have occurred, and accordingly, a mechanism for phosphate activation must have existed. Herein, we detail results of an investigation into prebiotic phosphate activation chemistry using simple, prebiotically available nitriles whose reactivity is increased by Cu^2+^ ions. Furthermore, although Cu^2+^ ions are known to catalyse the hydrolysis of phosphodiester bonds, we found this deleterious activity to be almost completely suppressed by inclusion of amino acids or dipeptides, which may suggest a productive relationship between protein and RNA from the outset.

## Abstract 2

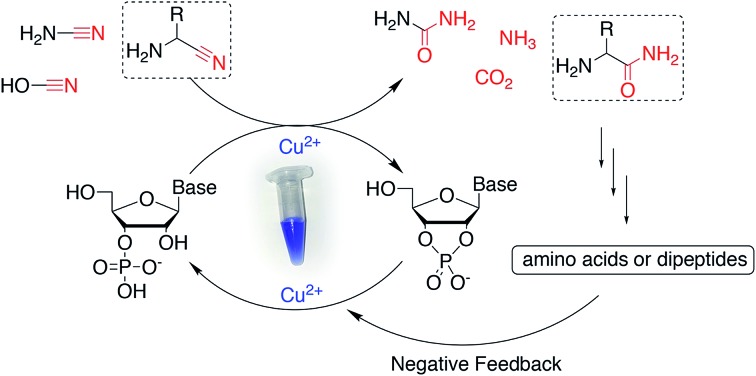
A synergistic system was established involving activating nucleotides with nitriles using Cu(ii) and protecting RNA degradation by byproducts of alpha-aminonitriles.

## Introduction

For over half a century the search for a simple prebiotic reagent which is capable of efficiently activating inorganic/nucleoside phosphate(s) has been largely unsuccessful.^1^ Yet, it stands to reason that an abiotic means of activating nucleotides (to allow replication by non-enzymatic polymerisation/ligation) was available on Earth in order to progress toward a more advanced chemical system. To circumvent this impasse, generally speaking, two approaches have been taken. In the first, *N*,*N*-dialkyl carbodiimides, not considered to have been available on early Earth but ubiquitous in synthetic chemistry, are used as phosphate activating agents, often at high concentration, in the presence or absence of other catalysts.^2^ A parallel has been drawn between *N*,*N*-dialkyl carbodiimides and carbodiimide (HN=C=NH), the tautomer of cyanamide, a presumed prebiotically abundant molecule.1*b* However, this tautomeric equilibrium lies overwhelmingly in favour of cyanamide, meaning that only trace amounts of carbodiimide are available for reaction with phosphate, which is reflected in the vast excesses of cyanamide that have to be employed to achieve moderate yielding but sluggish reactions.^3^ Consequently, arguments describing *N*,*N*-dialkyl carbodiimides as suitable prebiotic surrogates for cyanamide are not convincing. In the second approach, preformed (purified) phosphorimidazolides are used in the desired reaction, often with multiple rounds of addition thereof. Orgel found imidazole was a likely prebiotic molecule,^4^ produced by photochemical isomerization of β-aminoacrylonitrile.^5^ Furthermore, realising that nucleoside-5′-triphosphates are kinetically stable in the absence of enzyme catalysis, Orgel suggested that nucleophilic displacement of pyrophosphate from a nucleoside 5′-triphosphate by imidazole would give the corresponding phosphorimidazolide, a molecule with a similar free energy of hydrolysis to the parent triphosphate but also kinetically labile.^6^ Nucleoside 5′-phosphorimidazolides are known to be capable of efficiently extending a primer–template complex.^7^ However, absent from the literature is a prebiotically plausible, high-yielding, solution-phase synthesis of these activated monomers. The obvious requirement for prebiotic phosphate activation coupled with the obstinate problem of how prebiotic phosphate activation was achieved, is likely why the use of implausible activating agents and preformed phosphorimidazolides have been ‘tolerated’ in origins of life research – in order that ‘downstream’ prebiotic chemistry can be investigated. Current studies suggest that if non-enzymatic RNA replication was achieved *via* sequential monomer addition to a primer–template complex, phosphorimidazolides were required.^8^ If a convincing prebiotic synthesis of phosphorimidazolides cannot be found, it would suggest that non-enzymatic RNA replication was achieved *via* ligation of oligonucleotides on a template, as suggested by the work of von Kiedrowski.^9^ Furthermore, the chemistry which leads to activated (oligo)nucleotides could provide valuable insight into the geochemical scenario in which phosphate activation, and previous prebiotic synthesis, took place.

The early phase of high energy chemistry which must have taken place on primitive Earth, would be expected to generate a significant amount of small, multiple bond-rich molecules.^10^ If the potential energy locked in these multiple bonds could be harnessed, plentiful sources of prebiotic activating agents could have been available. This was recognized many years ago and was partly why molecules such as cyanate (NCO^–^), cyanogen ((CN)_2_) and cyanamide (NH_2_CN) were investigated as prebiotic activating agents.^1^ It is noteworthy that a large number of (proto)biomolecules are accessible in prebiotically plausible syntheses using, or producing, these same, small, high energy molecules.^10^,^11^ These chemical networks are consistent with a geochemical scenario.10b,11c,^12^ Given that these molecules are omnipresent in our protometabolic network, we were curious if their re-evaluation, in the context of our developing geochemical model, could provide a means to overcome inherent kinetic barriers and ‘switch on’ their reactivity by nitrile coordination. Thus, our attention turned to Fe^2+^, Fe^3+^, Ni^2+^, Cu^2+^, Co^2+^ and Zn^2+^ ions. Intriguingly, work from the Dronskowski group had shown that a variety of transition metal cations form complexes with cyanamide **1**. Being azophilic Cu^2+^ is expected to associate strongly with cyanamide **1**,^13^ but this is in contrast to oxophilic metal ions such as Mg^2+^. We began to examine the effect of these ions on reaction of adenosine 3′-phosphate (**2**, A3′P) with **1**.

## Results and discussion

Over the past few decades, nucleoside 3′-phosphates have been employed as a model system to study prebiotic phosphate activation chemistry.1a,^9^,^14^ In 1968, Orgel firstly recognized that transiently activated nucleoside 3′-phosphates would rapidly react intramolecularly with the adjacent nucleophilic 2′-OH group, consequently yielding nucleoside 2′,3′-cyclic phosphates (*e.g.* A > P **3**).1*a* More recently, we demonstrated the divergent reactivity of the transiently activated intermediate, as the 2′-OH group can attack either phosphorus or carbon, with the formation of the cyclic phosphate or a 2′-transferred product, respectively.^9^,14a,^15^ Hence, we began our study by investigating the effect of different metal ions (including Fe^2+^, Fe^3+^, Ni^2+^, Co^2+^, Cu^2+^ and Zn^2+^) on the cyanamide-induced activation of A3′P **2**. In a typical experiment, cyanamide **1** and **2** were incubated at 40 °C with, or without the metal ion, and the reaction was monitored after 20 h. While neither Fe^2+^ nor Fe^3+^ displayed any detectable catalytic effect on the cyclisation reaction and only a small improvement could be found when Zn^2+^ or Ni^2+^ or Co^2+^ were included in the mixture, Cu^2+^ efficiently promoted the formation of adenosine 2′,3′-cyclic phosphate (Table S1†). The catalytic effect of copper was significant even at concentrations as low as 1% relative to **1** (yield: 52% in 20 hours), whilst no competing 2′-transfer was observed under all the conditions tested. This cyanamide-Cu^2+^ system proved to be able to activate not only 3'-, but also 5′-phosphates, as demonstrated by the formation of adenosine pyrophosphate (A5′PP5′A, yield: 9%), following incubation of adenosine 5′-phosphate (A5′P) under similar conditions. But we have been unsuccessful in attempts to form the imidazolide of A5′P by *in situ* nitrile group activation chemistry. Whether this is either because imidazolides are not formed or because they are formed and then hydrolysed has not been investigated.

**Scheme 1:**
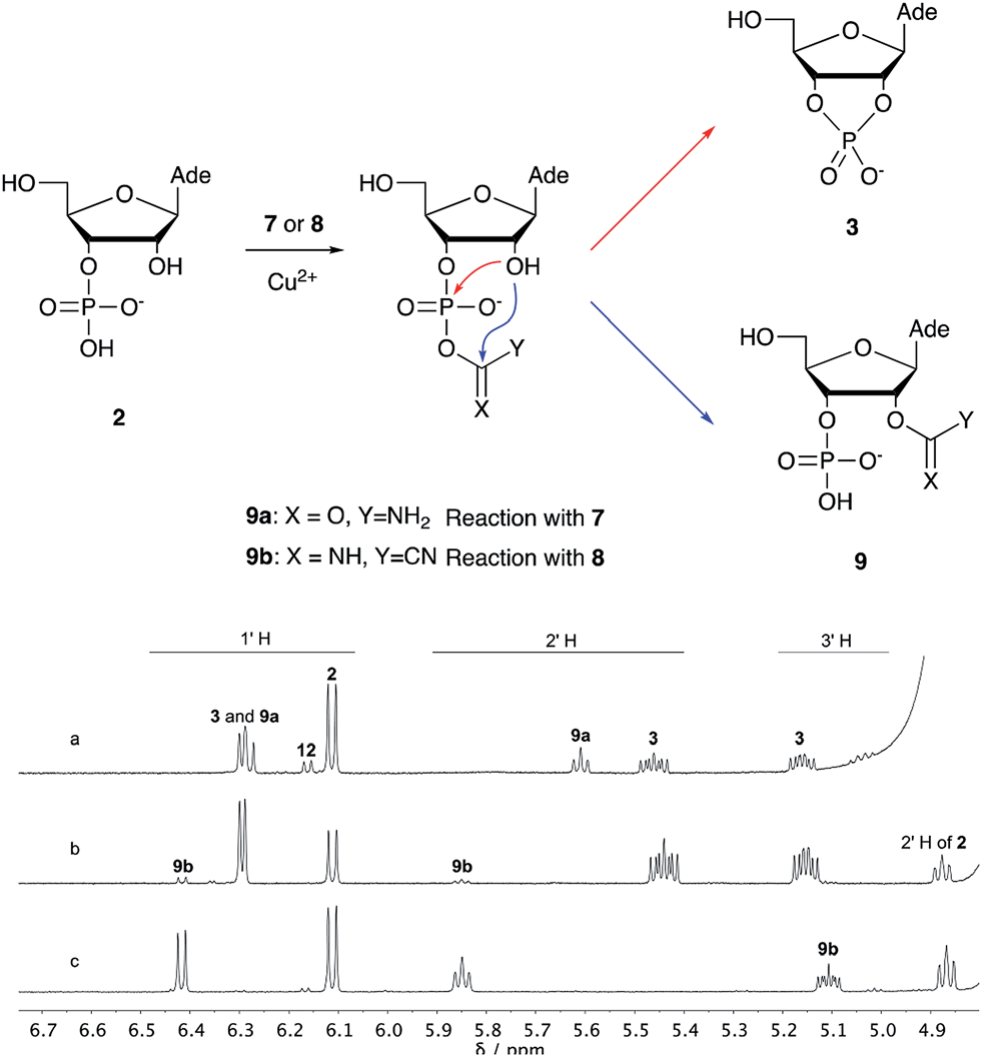
Mechanism of cyclisation *vs.* transfer in the cyanate- or cyanogen-mediated activation of **2** and ^1^H NMR spectra of the mixtures. (a) ^1^H NMR spectrum after 20 h following incubation of **2** (50 mM), CuCl_2_ (25 mM) and cyanate **7** (100 mM) at pH 4, 40 °C, showing the formation of **3** and **9a**; (b) ^1^H NMR spectrum after 1 h following incubation of **2** (12.5 mM), Gly (50 mM) CuCl_2_ (25 mM) and cyanogen **8** (100 mM) at pH 5.5, RT, showing the formation of **3** and **9b**; (c) as (b) but without CuCl_2_. N.B. conditions are different to Table 1.

Next, we investigated whether Cu^2+^ could catalyse phosphate activation using other prebiotically relevant nitrile-containing molecules (Table 1).^11^ Acetonitrile **4**, 3-aminopropionitrile **5** and glycolonitrile **6** were unsuccessful in promoting Cu(ii)-catalysed cyclization of A3′P **2**. Cyanate **7** and cyanogen **8** displayed a dual behaviour, producing **3** together with the 2′-adduct which will be discussed later (**9**, Scheme 1). Intriguingly, cyanogen **8** reacts with A3′P **2** even in the absence of a metal catalyst; however, the relative formation of the cyclised product and the 2′-transfer product is greatly affected by including Cu^2+^ in the mixture. And the reaction of A3′P with other nitriles in the absence of Cu^2+^ is insignificant.

**Table 1:** Cu^2+^-nitrile-mediated activation of A′3P and effect of Gly or GlyGly

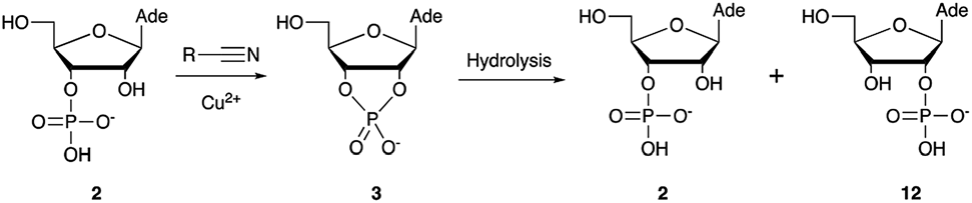								
Entry	Nitrile	R	Yield of 3 or 2′-transfer adducts^*a*^ (%)					
			CuCl_2_		CuCl_2_ and Gly		CuCl_2_ and GlyGly	
			**3** ^*b*^	2′-adduct	**3** ^*b*^	2′-adduct	**3** ^*b*^	2′-adduct
1	**1**	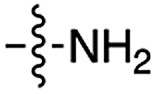	84	n.d.^*c*^	85^*d*^	n.d.	80	n.d.
2	**4**	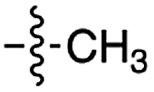	n.d.	n.d.	n.d.	n.d.	n.d.	n.d.
3	**5**	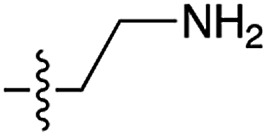	n.d.	n.d.	n.d.	n.d.	n.d.	n.d.
4	**6**	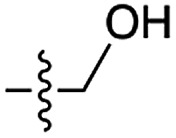	5	n.d.	2	n.d.	n.d.	n.d.
5	**7**	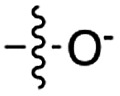	24	15	21	8	23	5.4
6	**8**	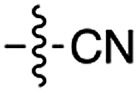	53	6	50	4	97^*e*^	3
7	**10**	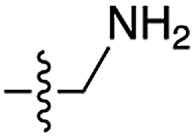	75	n.d.	60	n.d.	5	n.d.
8	**11**	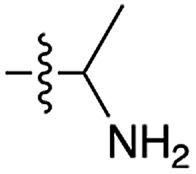	35	n.d.	22	n.d.	34	n.d.

^*a*^Standard reaction conditions: nitrile (100 mM), **2** (50 mM), CuCl_2_ (25 mM) and Gly or GlyGly (50 mM) in 90% H_2_O, 10% D_2_O at pH 4 (entries 1, 5 and 8) or pH 5.5 (entries 2, 3, 4, 6 and 7), heated at 40 °C for 20 hours.

^*b*^Inferred from the amount of **3** plus adenosine 2′-phosphate A2′P **12**, assuming that when **3** hydrolyses it always gives **2** and **12** in 1.8 : 1 ratios.^17^

^*c*^Not detected.

^*d*^Initial pH 5.5.

^*e*^Initial pH 4.

**Scheme 2:**
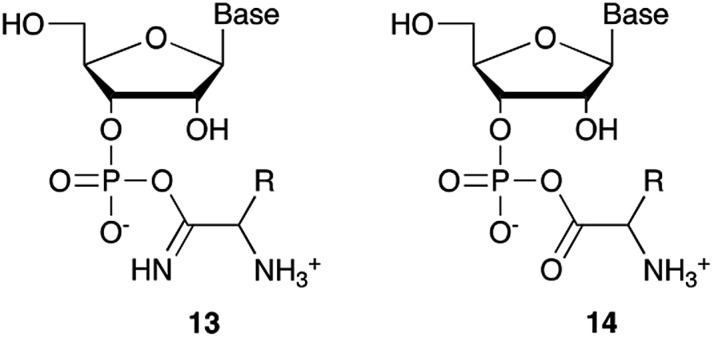
Structure of nucleoside 3′-phosphate imidoyl-**13** and carbonyl-**14** mixed anhydride derivatives.

We then turned our attention to aminoacetonitrile **10** and 2-aminopropionitrile **11**, the Strecker precursors of glycine and alanine, respectively, previously shown to originate from the same prebiotic pathways that form ribonucleotides, amino acids and phospholipid precursors.11*c* Interestingly, attack of a nucleoside monophosphate onto an α-aminonitrile would involve the formation of a transient imidoyl phosphate, analogous to the mixed anhydride produced by aminoacylation of nucleotides,14a,^15^ with the only difference being an imidoyl-**13** instead of a carbonyl-**14** derivative (Scheme 2). Based on the observation by Moureu and Bongrand^16^ that cuprous cyanoacetylide undergoes Glaser coupling^17^ to give dicyanodiacetylene on oxidation, we did not investigate Cu^2+^-catalysed addition of phosphates to cyanoacetylene as the related Eglinton reaction^18^ was anticipated.

Whilst mixed anhydride **14** has been previously been shown to both cyclise and give the 2′-adduct, both α-aminonitriles **10** and **11** triggered cyclisation of **2**, but the related 2′-transferred products were not detected. We speculate that the reactivity and/or the geometry of **13** could be altered by the simultaneous coordination of both the imido- and amino-nitrogen atoms to copper, somehow favouring cyclisation and hydrolysis over transfer. In this regard, we performed an experiment in which A5′P, aminoacetonitrile and Cu^2+^ were incubated in either H_2_^16^O, H_2_^18^O or a mixture of H_2_^16^O/H_2_^18^O (1 : 2), and monitored the isotopic composition of the products by mass spectrometry. In the absence of a vicinal hydroxyl group, activation of the 5′-phosphate of A5′P would result in the attack of water either on the activated phosphate or on the imidoyl-carbon of the imidoyl phosphate, producing ^18^O-labelled A5′P or ^18^O-labelled glycinamide, respectively (Scheme S1, S2 and Fig. S1†). In our system, glycinamide was the only new labelled product detected (the ratio of unlabelled/^18^O-labelled glycinamide was equal to the H_2_^16^O/H_2_^18^O ratio), thereby suggesting the selective attack of water on the imidoyl-carbon, and possibly a link with the aminoacyl-transfer chemistry described by Schimmel and co-workers on a minihelix.^19^

Optimization for cyclisation of A3′P **2** to **3** by modifying reaction conditions revealed that moderate to high yields could be obtained under slightly acidic conditions, but, alongside the expected cyclized product, we could detect the formation of adenosine 2′-phosphate (A2′P, **12**, Tables 1 and S2†).

Reasoning that the latter derived from hydrolysis of the former as the reaction progressed, we started examining the hydrolysis of **3** under these reaction conditions. As expected, Cu^2+^ catalysed the opening of the cyclic phosphate both at pH 4 and 5.5, producing **2** and **12** in 1.8 : 1 ratios.^20^ We thus wondered if ligands able to coordinate Cu^2+^ would attenuate the metal's hydrolytic activity. In particular, we focused our attention on prebiotically plausible chelating agents able to form bi- and tri-dentate complexes with copper ions,^21^ namely glycinamide, as the by-product of the aminoacetonitrile-mediated activation described above, its hydrolysis product glycine (Gly) and the dipeptide glycylglycine (GlyGly). The excellent coordinating properties of these ligands (Table S3†) considerably decreased the degree of cyclic phosphate hydrolysis, probably by competing with **3** for binding to the metal centre. In parallel, we examined urea and ammonium carbonate, the by-products of the cyanamide and cyanate-mediated activation, respectively, but, unsurprisingly, these failed to protect the cyclic phosphate from hydrolysis, presumably as a consequence of less favourable monodentate binding to copper.

Cu^2+^, as Mg^2+^, and other divalent metal ions, is also well known for its ability to catalyse RNA degradation,^22^ which proceeds through attack on the phosphodiester bond by the vicinal 2′-OH group, with formation of shorter 2′,3′-cyclic phosphate-terminated oligonucleotides (or a mixture of 2′- and 3′-monophosphates if the hydrolysis progresses further^23^). As a model for RNA degradation, we followed the hydrolysis of a 6-carboxyfluorescein (FAM)-labelled 10-mer RNA oligonucleotide incubated with CuCl_2_, in the presence or in the absence, of different amounts of Gly or GlyGly (60 °C, reactions monitored after 23 and 48 h). Whilst at pH 4, Cu^2+^ didn't affect the integrity of RNA (compared to buffer levels), at pH 5.2 and 7.0 (whereupon extensive precipitation of Cu(OH)_2_ occurred) the metal efficiently promoted RNA hydrolysis, with more than 96% and 57% degradation, respectively, after only 23 h. Remarkably, RNA was efficiently protected from Cu^2+^-catalysed degradation by addition of a small excess of Gly or GlyGly, despite the higher solubility of the metal under these conditions (Table S4 and Fig. S2–S6†). This is reminiscent of the demonstration that citrate protects RNA from Mg^2+^-catalysed degradation.^24^

We next tested the effect of these additives on the phosphate activation reaction, incubating **2**, cyanamide **1** and Cu^2+^ with either Gly or GlyGly. Both ligands suppressed the Cu^2+^-catalysed hydrolysis of the cyclic product, with the net effect of boosting the yield of **3** (Tables S5 and S6†). In the presence of Gly the activation of **2** to give **3** was highly efficient both at pH 4 and 5.5 (85% or 78%, respectively), however, with GlyGly the yields were reduced when the reaction was performed at pH 5.5 (37%). Likewise, the yields were negatively affected when the ligands were in high excess relative to copper. EPR spectroscopy of the Cu^2+^–GlyGly complex helped to elucidate the reasons for these outcomes. The coordination mode of GlyGly to Cu^2+^ is highly dependent on the pH of the solution, with the possibility of forming multiple species at equilibrium (over the pH range 4–7), including highly stable metal chelates.^21^ In agreement with previous literature,^25^ the EPR spectra of Cu^2+^–GlyGly mixtures at pH 4 mainly resembled the spectra of the aqua complex Cu(H_2_O)_6_^2+^, with smaller contributions from other species, presumably the two GlyGly bidentate complexes (Scheme S3, Fig S7 and Table S7†). Increasing the pH of the solution from 4 to 5.5 led to the almost complete disappearance of Cu(H_2_O)_6_^2+^ signal, and the spectra were dominated by the tridentate complex of Cu^2+^ and GlyGly (Fig. S7 and S8†). From these data, it is clear that as the pH of the mixture increases, the tridentate complex is formed at the expense of the cyanamide-induced activation of **2**. Thus, at high ligands concentrations, coordination of Gly and GlyGly to Cu^2+^ could saturate the metal ion coordination sphere, preventing cyclic phosphate and RNA hydrolysis. However, the competitive binding of **1** to Cu^2+^ in the presence of these ligands is still possible. The available data do not allow a detailed picture of the mechanism, although we suspect that there are several catalytically active complexes. Further investigation was not made as our interests were with the conversion *per se*. With the possibility of small alterations in the reaction conditions perturbing yields and product distribution, we explored the cyanamide **1**-Cu^2+^-mediated nucleotide activation over a range of temperatures (Fig. S9†), pHs and concentrations, obtaining the maximum yields of 84% and 90% of **3** (other nucleotides shown in Table S8†) when the reaction was performed with **1** (100 mM), **2** (12.5 mM) and CuCl_2_ (25 mM) in the presence of Gly (pH 5.5, 50 mM, 40 °C) or GlyGly (pH 4, 12.5 mM, 40 °C), respectively. It was found that multiple equivalents of cyanamide are required for optimum yield, we assume that water competes with phosphate in the attack on metal coordinated cyanamide with the result that multiple equivalents of urea are formed as a by-product.

## Conclusions

**Scheme 3:**
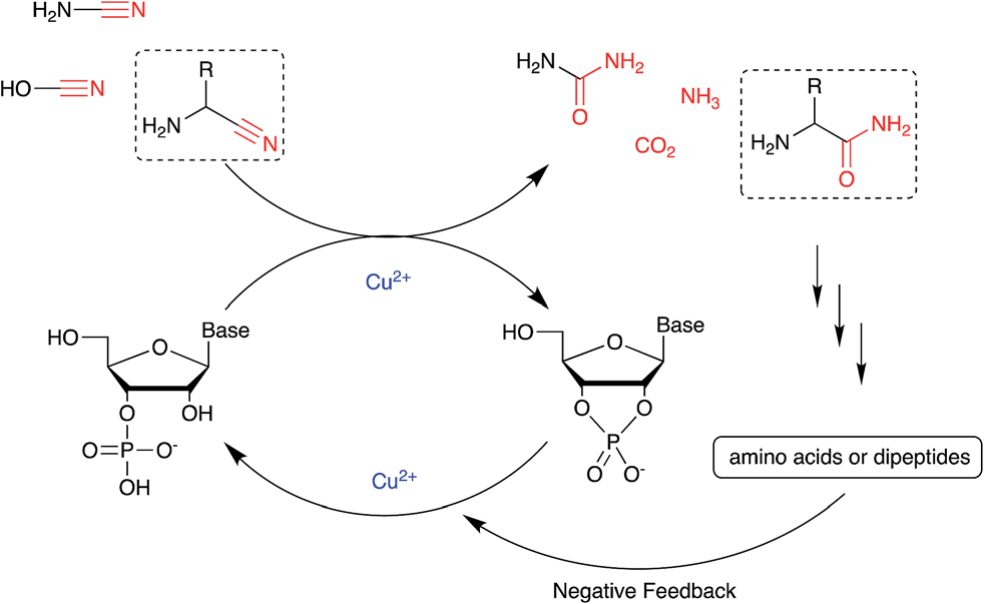
A synergistic system including activation and protection chemistry.

Nucleotide activation has been a central problem in prebiotic chemistry for nearly 60 years. Orgel1*a* first showed uridine-2′,3′-cyclic phosphate was generated under harsh conditions, using high concentration of cyanamide and heating for a long time. Then we found α-ketoacids catalyse cyclic phosphate formation with cyanamide,14c and this prompted our search for other catalysts in particular ones which would work with other nitriles. In the present report, we show how Cu^2+^ ions efficiently trigger the reactivity of prebiotically plausible nitriles, providing a means to unlock the energy stored in these molecules and efficiently use it in the context of nucleotide activation chemistry. Particularly intriguing is the discovery that aminoacetonitrile, available from the same prebiotic pathways that produce ribonucleotides,11*c* is able to promote activation of nucleotides. The by-product of this reaction is glycinamide, which upon (Cu^2+^-catalysed^26^) hydrolysis would deliver glycine into the environment, establishing a further link between the nucleotide and the amino acid sub-systems. In parallel, we found that glycinamide, glycine and the dipeptide (GlyGly) are needed to suppress the detrimental role that Cu^2+^ ions exert in promoting the hydrolysis of cyclic phosphates and RNA phosphodiester bonds. A synergistic connection between activation and protection chemistry is therefore evident, and suggestive of a scenario in which progressive cycles of activation and glycinamide hydrolysis will feed the glycine pool (and eventually produce GlyGly under coupling conditions), thereby preventing hydrolysis and enabling the accumulation of short oligonucleotides (Scheme 3).

## Conflicts of interest

There are no conflicts to declare.

## Supplementary Material

Supplementary information
