# Supplementary material for: Tuning the reactivity of nitriles using Cu(ii) catalysis – potentially prebiotic activation of nucleotides
Source: Chem Sci. 2018 Jul 25;9(35):7053–7. doi: 10.1039/c8sc02513d (PMC6137443; doi:10.1039/c8sc02513d)
Supplement: Supplementary file 1 [file SC-009-C8SC02513D-s001.pdf]

Electronic Supplementary Material (ESI) for Chemical Science.
This journal is © The Royal Society of Chemistry 2018

Supporting Information

Tuning the reactivity of nitriles using Cu(II) catalysis – potentially prebiotic activation of nucleotides

Ziwei Liu,[a] Angelica Mariani,[a] Longfei Wu,[a] Dougal Ritson,[a] Andrea Folli,[b] Damien Murphy[b] and John Sutherland*[a]

a. MRC Laboratory of Molecular Biology, Francis Crick Avenue, Cambridge Biomedical Campus, CB2 0QH, UK.

b. School of Chemistry, Cardiff University, Park Place, Cardiff CF10 3AT, U.K.

** Email: johns@mrc-lmb.cam.ac.uk.*

1. Abbreviation list

Acetonitrile	ACN
Adenosine 2':3'-cyclic monophosphate	A>P
Adenosine 2'-monophosphate	A2'P
Adenosine 2'& 3'-monophosphate mixture	A2'3'P
Adenosine 3'-monophosphate	A3'P
Adenosine 5'-monophosphate	A5'P
Adenosine pyrophosphate	AppA
Aminoacetonitrile	AAN
2-Aminopropionitrile	2-APN
3-Aminopropionitrile	3-APN
6-carboxyfluorescein	FAM
Cytidine 3'-monophosphate	C3'P
Glycine	Gly
Glycylglycine	GlyGly
Guanosine 2' & 3'-monophosphate mixture	G2'3'P
Triethylamine	TEA

2. General Methods

Reagents and solvents were obtained from *Acros Organics*, *Alfa Aesar*, *Santa Cruz Biotechnology*, *Sigma-Aldrich* and *VWR International*, and were used without further purification unless otherwise stated. The FAM-10nt RNA oligonucleotide (5'(6-FAM)-UGUGCCAGUA-3') was purchased in the HPLC-purified Na^+ form from *Integrated DNA Technologies*. ^{18}O-Labelled water had an isotopic incorporation of 97%. A *Mettler Toledo* SevenEasy pH Meter S20 combined with a *ThermoFisher Scientific* Orion 8103BN Ross semi-micro pH electrode was used to measure and adjust the pH to the desired value. ^1H, ^{31}P, and ^{13}C nuclear magnetic resonance (NMR) spectra were acquired using a *Bruker* Ultrashield 400 Plus or *Bruker* Ascend 400 operating at 400.13, 161.97, and 100.62 MHz, respectively. Samples consisting of H_2O/D_2O mixtures were analyzed using HOD suppression to collect ^1H NMR spectroscopy data. Coupling constants (J) are given in Hertz and the notations s, t, and br s represent the multiplicities singlet, triplet, and broad signal, respectively. Chemical shifts (δ) are shown in ppm. Mass spectra were acquired on an *Agilent* 1200 LC-MS system equipped with an electrospray ionization (ESI) source and a 6130 quadrupole spectrometer (LC solvents: A, 0.2 % formic acid in H_2O – B, and 0.2 % formic acid in ACN). Gel electrophoresis: 20 % polyacrylamide, 8 M urea gels (0.75 mm thick, 20 cm long) were typically run at 15 W in TBE buffer. Fluorescence imaging was performed using an Amersham Typhoon imager (*GE healthcare*) and quantified using ImageQuant TL software (version 7.0). The continuous wave electron paramagnetic resonance (CW-EPR) spectra were measured on a Bruker EMX spectrometer using a Bruker ER4119HS resonator operating at 120 K, 100 kHz field modulation frequency, 3.0 G field modulation amplitude, 6.4 mW microwave power and 10^3 receiver gain (full spectra) or 1.42×10^4 receiver gain (magnification of the parallel component of the Cu **g** tensor). For all the EPR samples, the solvent used was a mixture 50:50 H_2O/glycerol. This was necessary in order to obtain a good glass for EPR measurements of frozen solutions at 120 K. Simulations were performed using the Easyspin software package[S1] running within the MathWorks MatLab environment.

3. Experimental procedures

3.1 General procedure to remove copper before NMR spectroscopy

In order to prepare the sample for NMR analysis, a solution of NaHS in water (1 M, 2 equiv relative to copper) was added to the mixture at the end of the reaction. The pH of the solution was adjusted to 5 – 5.5 by addition of a 1 M solution of HCl in water. The resulting black precipitate was removed by centrifugation. D_2O (0.1 mL) was added to the supernatant and the mixture was analysed by ^1H and ^{31}P NMR.

3.2 General procedure for the reaction with cyanamide (see Table S1, S2, S5 and S6 for details)

A3'P (12.5 mM or 50 mM) was dissolved in water, and the pH of the solution was adjusted to the desired value with HCl (1 M) or NaOH (1 M) solutions. In parallel, a solution of the metal salt (25 mM) was prepared by adding the desired amount of Gly or GlyGly, and by adjusting the pH to the same value of the nucleotide solution. The two solutions were then mixed together and the desired amount of cyanamide was added. The resulting mixture was incubated at 40 °C for 20 hours. The reaction was analysed by NMR spectroscopy, after copper removal.

3.3 General procedure for the reaction with the nitriles 4, 5, 6, 7, 10 and 11 (see Table 1 and Scheme 1 for details)

A3'P (50 mM) and the desired nitrile were dissolved in water, and the pH of the solution was adjusted to the desired value with HCl (1 M) or NaOH (1 M) solutions. In parallel, a solution of $CuCl_2$ (25 mM) was prepared by adjusting the pH to the same value of the A3'P solution. The two solutions were then mixed together and incubated at 40 °C for 20 hours. The reaction was analysed by NMR spectroscopy, after copper removal.

3.4 Synthesis and isolation of cyanogen

To a 3-necked round-bottomed flask containing a solution of $CuCl_2 \cdot 2H_2O$ (50.0 mmol, 8.53 g) in H_2O (9 mL) and a stirrer bar was connected an N_2 inlet (used as a carrier gas), a small dropping funnel and a gas outlet. The gases flowing through the outlet were sparged through an aqueous solution (25 mL) of $Ag(NO_3)$ (25.0 mmol, 4.25 g) with 2 drops of 2 M nitric acid added, then passed through a drying column containing $CaCl_2$ and finally through a -78 °C trap. The effluent gases were scrubbed through a sodium hydroxide/bleach solution. In parallel, NaCN (1.04 equiv, 51.8 mmol, 2.54 g) was dissolved in the minimum volume of H_2O (*ca.* 5 mL). The resulting solution was charged to the dropping funnel and allowed to drop into the stirred copper solution at a rate such that the addition took *ca.* 45 min. The reaction was mildly exothermic, with evolution of gas (the rate of cyanide addition was slowed if the reaction became too aggressive/warm). At the end of the cyanide addition, sufficient cyanogen (colourless solid in the final trap) was deemed to have been collected. Hence, the remaining cyanogen dissolved in the copper and in the $Ag(NO_3)$ solutions was sparged directly through the bleach solution (although this could have been collected if desired). The trap was stoppered with a new septum and an empty balloon attached via a needle. The flask was removed from the acetone/CO_2(s) bath and HPLC grade ACN (1.24 g, 1.57 mL) added and the cyanogen dissolved in it. The flask was placed in an ice/water bath. A small flask was stoppered with a new septum and pre-weighed, then an empty balloon was attached via needle. The cyanogen solution was syringed into the small flask, balloon removed and weighed. It was determined that cyanogen (1.92 mmol, 100 mg, 8 %) had been recovered which gave a solution of *ca.* 1.2 M cyanogen in ACN.

3.5 General procedure for the reaction with cyanogen (see Table 1 and Scheme 1 for details)

A3'P (12.5 mM or 50 mM) and the desired amount of Gly and $CuCl_2$ (if any) were dissolved in water, and the pH of the solution was adjusted the desired value with HCl (1 M) or NaOH (1 M) solutions. A solution of cyanogen in ACN (1.2 M, 50 ul) was then added. The mixture was kept at room temperature for 1 or 20 hours. The reaction was analysed by NMR spectroscopy, after copper removal (if needed).

3.6 Formation of AppA from the reaction of A5'P with cyanamide-Cu^{2+}

A5'P (50 mM) and $CuCl_2$ (25 mM) were dissolved in water, and the pH of the solution was adjusted to 5.5 with HCl (1 M) or NaOH (1 M) solutions. Cyanamide (100 mM) was added, and the mixture was incubated at 40 °C for 20 hours. The reaction was analysed by NMR spectroscopy, after copper removal.

3.7 Reaction of A5'P with AAN-Cu^{2+} in $H_2{}^{18}O$

A5'P (25 mM), AAN hydrochloride salt (250 mM) and $CuCl_2$ (50 mM) were dissolved either in water, ^{18}O-labelled water or a mixture of $H_2{}^{16}O/H_2{}^{18}O$ (1:2), and the pH of the solution was adjusted to 5.5 with HCl (1 M, $H_2{}^{16}O$ or $H_2{}^{18}O$ solution) or NaOH (1 M, $H_2{}^{16}O$ or $H_2{}^{18}O$ solution). The mixtures were incubated at 40

°C over the weekend and then analysed by ESI-MS, after copper removal. Since AAN couldn't be detected under these conditions, AAN was derivatised as follows. After the activation reaction and copper removal, an aliquot of the resulting solution was lyophilized and then resuspended in THF (containing 1 equiv of TEA relative to AAN). Acetyl chloride (2 equiv relative to AAN) and TEA (1 equiv) were then added and the mixture was kept at room temperature for 2 hours. The reaction was analysed by ESI-MS, after dilution with water.

3.8 General procedure to study oligonucleotides hydrolysis

Hydrolysis buffers (see Table S5 for details): a stock solution of $CuCl_2$ (20 mM) was pre-mixed with the desired amount of either Gly (200 mM) or GlyGly (200 mM) and then diluted with H_2O and the appropriate buffer (0.5 M sodium acetate pH 5 or 0.5 M HEPES pH 7). The pH of the solutions was then adjusted to the desired value by addition of HCl/NaOH solutions. Precipitation of $Cu(OH)_2$ was observed at pH 7 only in the absence of the Gly/GlyGly ligands.

Degradation experiment: 5 µL of each hydrolysis buffer was independently mixed with 5 µL of a FAM-10nt oligonucleotide 40 µM solution (final concentrations halved compared to the original solutions). The reactions were kept at 60 °C for 48 h and their progress was monitored after 23 and 48h. Aliquots of 1 µL were taken at the appropriate time, diluted with 9 µL of loading buffer (95 % (v/v) formamide, 5 % (v/v) glycerol, 50 mM EDTA, 0.5 mg/ml Orange G) and analysed by gel electrophoresis.

4. Supplementary Tables and Figures

Table S1. Yield % of A>P following reaction of A3'P with cyanamide in the presence of different transition metal ions. Standard reaction conditions: A3'P (50 mM), cyanamide (100 mM) and metal salt (50 mM) in water, at 40 °C for 20 hours, initial pH 5.5.

**Metal salts**	$CuCl_2$	$NiCl_2$	$CoCl_2$	$ZnCl_2$	$FeSO_4$	$FeCl_3$	-[a]
Yield of A>P (%)	60.0	10.8	7.7	4.0	<1.0	<1.0	<1.0

a. without any metal salts added.

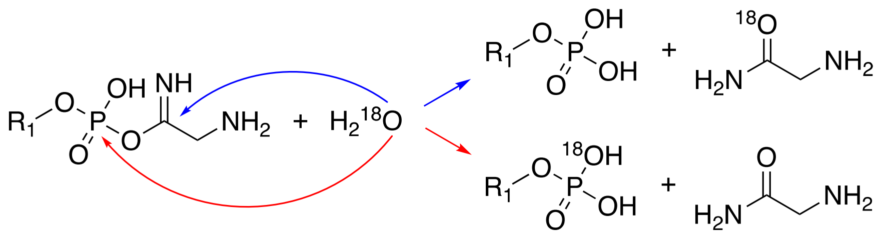

Scheme S1. Mechanism of the two possible hydrolysis pathways for the transient *O*-phosphoryl imidate that is obtained from the reaction of A5'P with AAN-Cu^{2+} in $H_2{}^{16}O/H_2{}^{18}O$ (1:2).

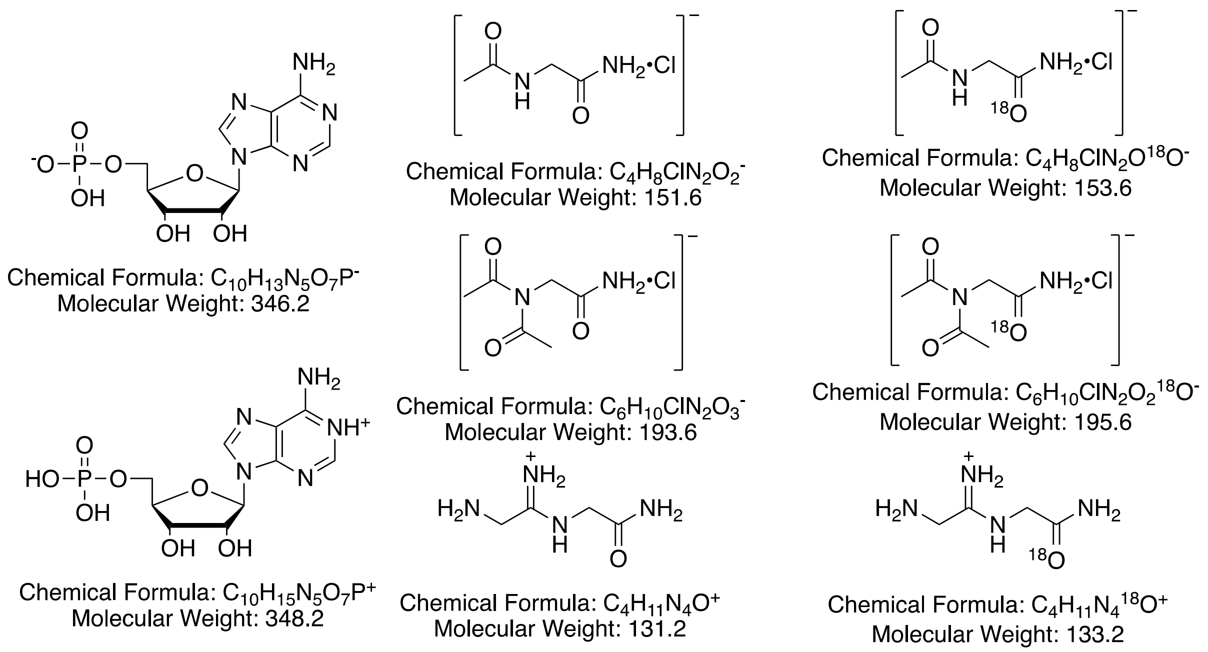

Scheme S2. Structure of the products obtained from the reactions of A5'P with AAN-Cu^{2+} in $H_2{}^{16}O/H_2{}^{18}O$ (1:2).

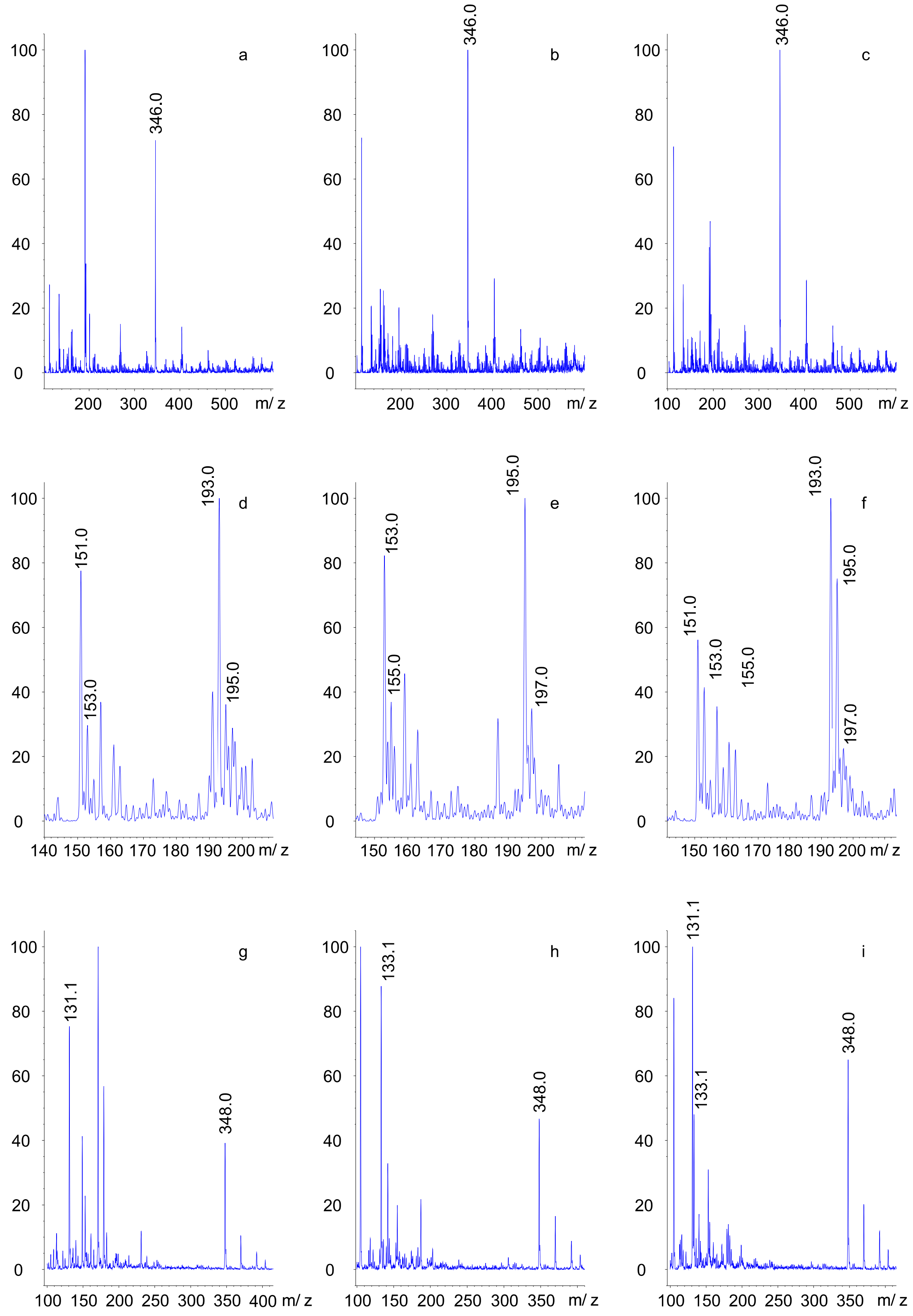

Fig. S1. Mass spectra of the reaction of A5'P with AAN-Cu^{2+} in either water, ^{18}O-labelled water or a mixture of $H_2{}^{16}O/H_2{}^{18}O$ (1:2). A-F) Negative mode; A) reaction in unlabelled water; B) reaction in ^{18}O-labelled water; C) reaction in $H_2{}^{16}O/H_2{}^{18}O$ (1:2); D) reaction in unlabelled water, after AAN derivatization

with acetyl chloride; E) reaction in ^{18}O-labelled water, after AAN derivatization with acetyl chloride; F) reaction in $H_2{}^{16}O/H_2{}^{18}O$ (1:2), after AAN derivatization with acetyl chloride. G-I) Positive mode, G) reaction in unlabelled water; H) reaction in ^{18}O-labelled water; I) reaction in $H_2{}^{16}O/H_2{}^{18}O$ (1:2).

Table S2. Product distribution (%) for A3'P, A>P and A2'P and overall A>P yield %, following incubation of A3'P with cyanamide-Cu^{2+}, at different pH values and different cyanamide concentrations. Standard reaction conditions: A3'P (50 mM), $CuCl_2$ (25 mM), pH and cyanamide as indicated, 40 °C for 20 hours.

pH value	Cyanamide (mM)	A>P	A2'P	A3'P	Overall A>P
pH 4	25	20.2 %	6.8 %	73.0 %	39.3 %
	50	34.6 %	7.6 %	57.8 %	55.8 %
	75	33.5 %	14.7 %	51.8 %	74.6 %
	100	45.0 %	14.0 %	41.0 %	84.2 %
	125	31.1 %	20.1 %	48.8 %	87.4 %
	150	29.4 %	22.6 %	48.1 %	92.6 %
pH 5	25	12.8 %	1.8 %	85.4 %	17.9 %
	50	25.4 %	1.5 %	73.2 %	29.5 %
	75	39.4 %	1.3 %	59.3 %	43.0 %
	100	49.1 %	2.2 %	48.7 %	55.4 %
	125	60.0 %	2.3 %	37.7 %	66.4 %
	150	59.9 %	2.0 %	38.1 %	65.5 %
pH 6	25	7.9 %	1.5 %	90.7 %	12.0 %
	50	16.4 %	1.3 %	82.4 %	19.9 %
	75	21.5 %	1.0 %	77.5 %	24.3 %
	100	23.7 %	1.3 %	75.0 %	27.3 %
	125	24.5 %	1.1 %	74.4 %	27.6 %
	150	24.2 %	1.4 %	74.4 %	28.0 %

Table S3. Product distribution (%) for A3'P and A2'P following incubation of A>P under different conditions. Standard reaction conditions: A>P (25 mM), $CuCl_2$ (12.5 mM), 40 °C for 3 hours.

Additive	pH 4		pH 5.5	
	A3'P	A2'P	A3'P	A2'P
-[a]	9.1 %	3.0 %	21.0 %	8.2 %
Urea (25 mM)	8.2 %	2.8 %	22.3 %	6.8 %
NH_4HCO_3 (25 mM)	6.6 %	2.1 %	19.1 %	7.6 %
Glycinamide (25 mM)	5.7 %	1.7 %	3.6 %	1.5 %
Gly (25 mM)	4.2 %	1.5 %	1.2 %	0.7 %
GlyGly (25 mM)	6.3 %	2.2 %	Trace	Trace
Control experiment[b]	Trace	Trace	Trace	Trace

a. $CuCl_2$ was the only component.

b. Hydrolysis of A>P without $CuCl_2$ or other components.

Table S4. Components, concentration and pH values of the hydrolysis buffers used in the RNA

degradation experiments.

	$CuCl_2$ (mM)	Ligand (mM)	Buffer (100mM)	pH
Cu(II) controls	40	0	AcONa/AcOH	4.0 5.2
			HEPES	7.0
Gly-containing buffers	40	4	AcONa/AcOH	4.0 5.2
			HEPES	7.0
		12	AcONa/AcOH	4.0 5.2
			HEPES	7.0
		40	AcONa/AcOH	4.0 5.2
			HEPES	7.0
GlyGly-containing buffers	40	4	AcONa/AcOH	4.0 5.2
			HEPES	7.0
		8	AcONa/AcOH	4.0 5.2
			HEPES	7.0
		20	AcONa/AcOH	4.0 5.2
			HEPES	7.0
		40	AcONa/AcOH	4.0 5.2
			HEPES	7.0
Buffer controls	0	0	AcONa/AcOH	4.0 5.2
			HEPES	7.0

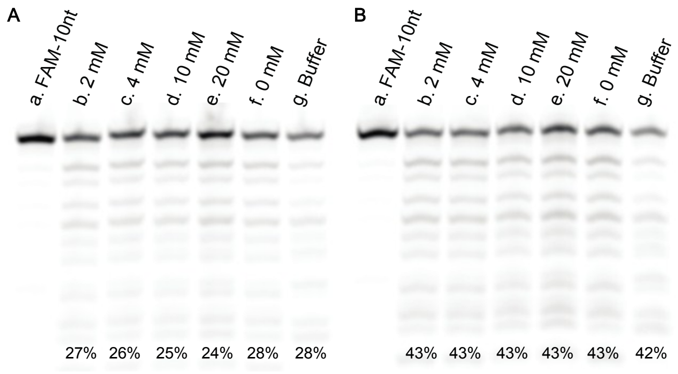

Fig. S2. A) Degradation of the FAM-10nt oligonucleotide at pH 4.0 in GlyGly-containing hydrolysis buffers after 23h. B) as A) after 48h. Lane a, standard; b-e, $CuCl_2$ 2mM, AcONa/AcOH 50mM, GlyGly 2, 4, 10, 20 mM, respectively; f, $CuCl_2$ 2mM, AcONa/AcOH 50mM; g, AcONa/AcOH 50mM. Degradation yields as labelled on each lane.

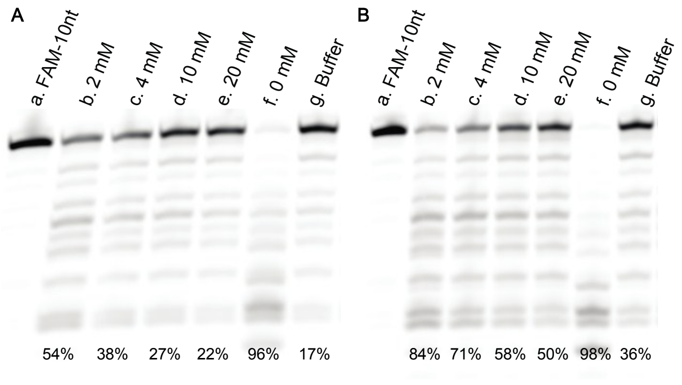

Fig. S3. A) Degradation of the FAM-10nt oligonucleotide at pH 5.2 in GlyGly-containing hydrolysis buffers after 23h. B) as A) after 48h. Lane a, standard; b-e, $CuCl_2$ 2mM, AcONa/AcOH 50mM, GlyGly 2, 4, 10, 20 mM, respectively; f, $CuCl_2$ 2mM, AcONa/AcOH 50mM; g, AcONa/AcOH 50mM. Degradation yields as labelled on each lane.

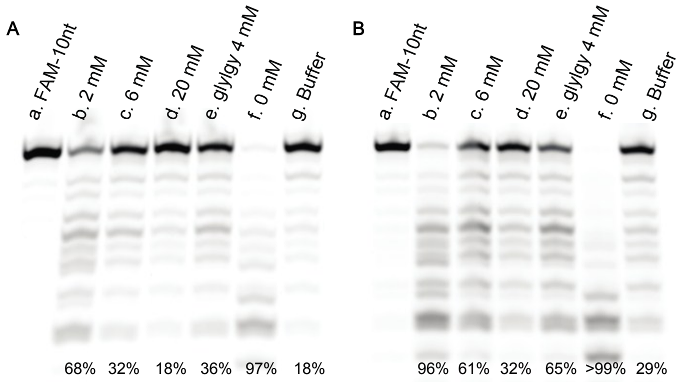

Fig. S4. A) Degradation of the FAM-10nt oligonucleotide at pH 5.2 in Gly-containing hydrolysis buffers after 23h. B) as A) after 48h. Lane a, standard; b-d, $CuCl_2$ 2mM, AcONa/AcOH 50mM, Gly 2, 6, 20 mM, respectively; e, $CuCl_2$ 2mM, AcONa/AcOH 50mM, G-G 4 mM; f, $CuCl_2$ 2mM, AcONa/AcOH 50mM; g, AcONa/AcOH 50mM. Degradation yields as labelled on each lane.

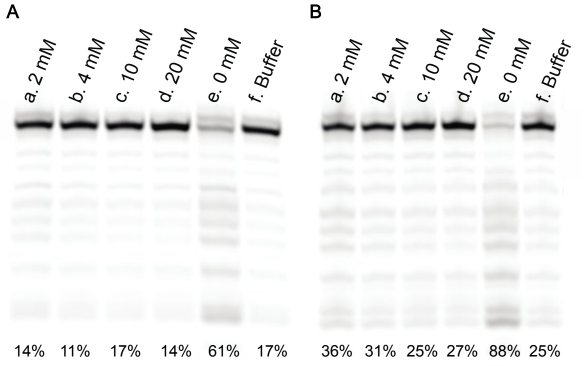

Fig. S5. A) Degradation of the FAM-10nt oligonucleotide at pH 7.0 in GlyGly-containing hydrolysis buffers after 23h. B) as A) after 48h. Lane a-d, $CuCl_2$ 2mM, HEPES 50mM, GlyGly 2, 4, 10, 20 mM, respectively; e, $CuCl_2$ 2mM, HEPES 50mM; f, HEPES 50mM. Degradation yields as labelled on each lane.

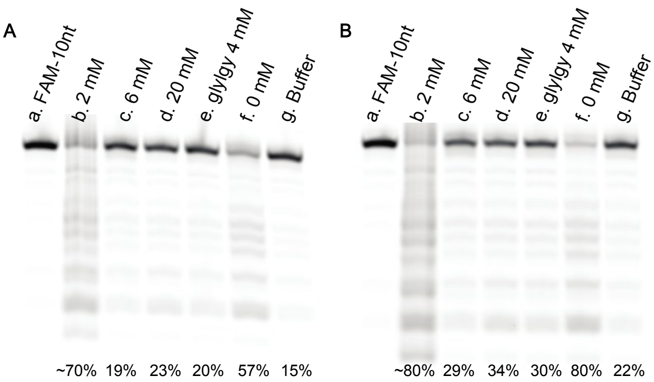

Fig. S6. A) Degradation of the FAM-10nt oligonucleotide at pH 7.0 in Gly-containing hydrolysis buffers after 23h. B) as A) after 48h. Lane a, standard; b-d, $CuCl_2$ 2mM, HEPES 50mM, Gly 2, 6, 20 mM, respectively; e, $CuCl_2$ 2mM, HEPES 50mM, G-G 4 mM; f, $CuCl_2$ 2mM, HEPES 50mM; g, HEPES 50mM. Degradation yields as labelled on each lane.

Table S5. Product distribution (%) for A3'P, A>P and A2'P and overall A>P yield %, following incubation of A3'P with Gly and cyanamide-Cu^{2+}, at different pH values and different Gly concentrations. Standard reaction conditions: A3'P (12.5 mM), $CuCl_2$ (25 mM), cyanamide (100 mM), pH and Gly as indicated, 40 °C for 20 hours.

pH value	Gly (mM)	A>P	A2'P	A3'P	Overall A>P
pH 4	25	69.4 %	5.7 %	25.0 %	85.2 %
	50	55.4 %	1.7 %	43.0 %	60.0 %
	75	41.1 %	<0.1 %	58.9 %	41.1 %
	100	31.9 %	1.0 %	67.1 %	34.7 %
pH 5.5	25	71.8 %	2.3 %	25.9 %	78.2 %
	50	83.5 %	1.5 %	15.0 %	87.7 %
	75	63.4 %	<0.1 %	36.6 %	63.4 %
	100	49.5 %	<0.1 %	50.5 %	49.5 %

Table S6. Product distribution (%) for A3'P, A>P and A2'P and overall A>P yield %, following incubation of A3'P with GlyGly and cyanamide-Cu^{2+}, at pH 4 and different GlyGly concentrations. Standard reaction conditions: A3'P (12.5 mM), $CuCl_2$ (25 mM), cyanamide (100 mM), pH 4 and GlyGly as indicated, 40 °C for 20 hours.

Concentration of GlyGly (mM)	A>P	A2'P	A3'P	Overall A>P
12.5	89.6 %	1.3 %	9.1 %	93.1 %
25	87.8 %	1.6 %	10.6 %	92.3 %
50	79.4 %	1.0 %	19.6 %	82.1 %
125	55.1 %	0.6 %	44.3 %	56.8 %
250	34.2 %	<0.1 %	65.8 %	34.2 %

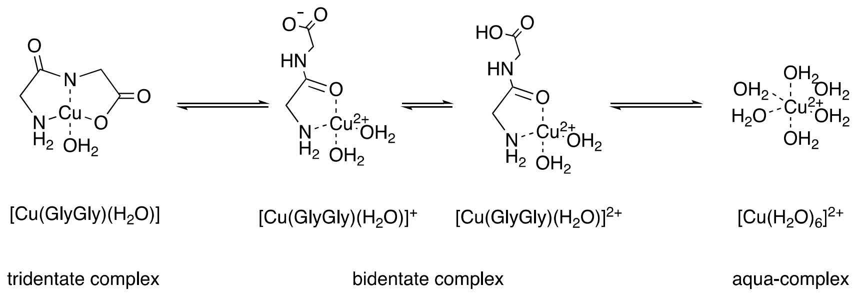

Scheme S3. GlyGly coordination modes to Cu^{2+} in water, at different pH values.

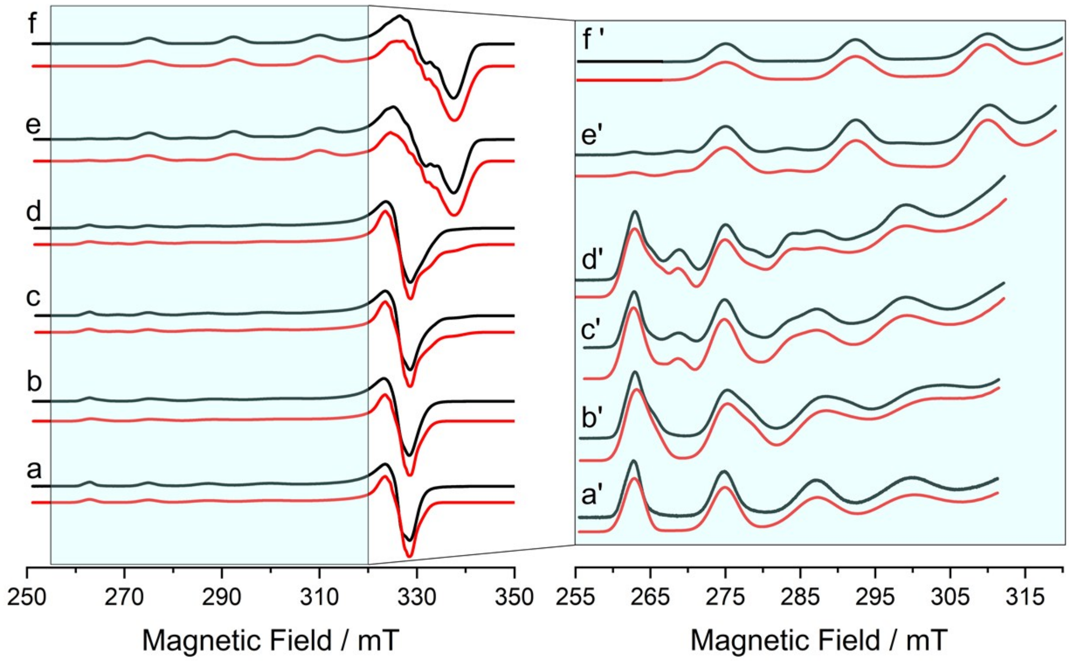

Fig. S7. X-band (9.5 GHz) continuous wave(CW)-EPR spectra (black traces) and simulations (red traces) of (a, a') $CuCl_2$ 10 mM at pH 4, consisting of 100 % of $[Cu(H_2O)_6]^{2+}$. (b, b') $CuCl_2$ 10 mM + H_2NCN 40 mM at pH 4 consisting of 66.(6) % spectral contribution of $[Cu(H_2O)_6]^{2+}$ and 33.(3) % spectral contribution of $[Cu(H_2NCN)(H_2O)_3]^{2+}$. (c, c') Cu(GlyGly) complex[S2] 10 mM at pH 4 consisting of 71.4 % spectral contribution of $[Cu(H_2O)_6]^{2+}$, 17.9 % spectral contribution of $[Cu(GlyGly)(H_2O)_2]^{+}$ and 10.7 % spectral contribution of $[Cu(GlyGly)(H_2O)_2]^{2+}$. (d, d') $CuCl_2$ 12.5mM + GlyGly 25 mM at pH 4 consisting of 56.2 % spectral contribution of $[Cu(H_2O)_6]^{2+}$, 21.9 % spectral contribution of $[Cu(GlyGly)(H_2O)_2]^{+}$ and 21.9 % spectral contribution of $[Cu(GlyGly)(H_2O)_2]^{2+}$. (e, e') Cu(GlyGly) complex 10 mM at pH 5.5 consisting of 8.(3) % spectral contribution of $[Cu(H_2O)_6]^{2+}$, 8.(3) % spectral contribution of $[Cu(GlyGly)(H_2O)_2]^{+}$ and 83.(3) % spectral contribution of $[Cu(GlyGly)(H_2O)]$. (f, f') Cu(GlyGly) complex 10 mM at pH 7 consisting of 100 % of $[Cu(GlyGly)(H_2O)]$.

Table S7. Spin Hamiltonian parameters for the different Cu^{2+} species identified from the EPR spectra shown in Figure S7. The Euler angles associated with the rotation of the ***g*** and ^{Cu}***A*** tensors with respect to the molecular frame are given by α, β, γ for the ***g*** and ^{Cu}***A*** tensor frames.

species	g_x	g_y	g_z	α	β	γ	A_x	A_y	A_z	α	β	γ
				rad	rad	rad	MHz	MHz	MHz	rad	rad	rad
					g frame						***A*** frame	
$[Cu(H_2O)_6]^{2+}$	2.075*	2.086*	2.416*	-1.51	1.61	2.97	-27#	-25#	-400$	1.63	1.59	-1.52
$[Cu(GlyGly)(H_2O)_2]^{+}$	2.045*	2.053*	2.338*	-1.41	1.66	2.90	-27#	-25#	-465$	1.63	1.59	-1.52
$[Cu(GlyGly)(H_2O)_2]^{2+}$	2.052*	2.083*	2.378*	-1.61	1.71	2.87	-27#	-25#	-440$	1.63	1.59	-1.52
$[Cu(GlyGly)(H_2O)]$	2.051*	2.056*	2.255*	-1.51	1.61	2.97	-35#	-36#	-535$	1.63	1.59	-1.52
$[Cu(H_2NCN)(H_2O)_3]^{2+}$	2.075*	2.086*	2.382*	-1.51	1.61	2.97	-27#	-25#	-430$	1.63	1.59	-1.52

* ± 0.003; # ± 5 MHz; $ ± 3 MHz.

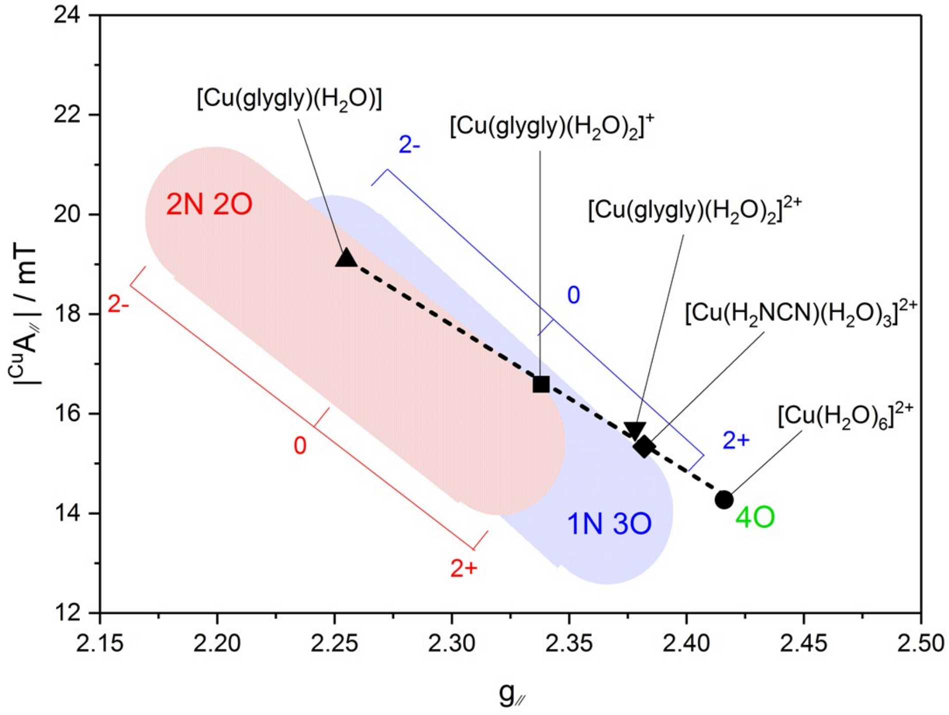

Fig. S8. Peisach-Blumberg plot of $^{Cu}A_{//}$ vs $g_{//}$ of the five species identified by the spectra in Fig. S7 with spin Hamiltonian parameters listed in Table S7.

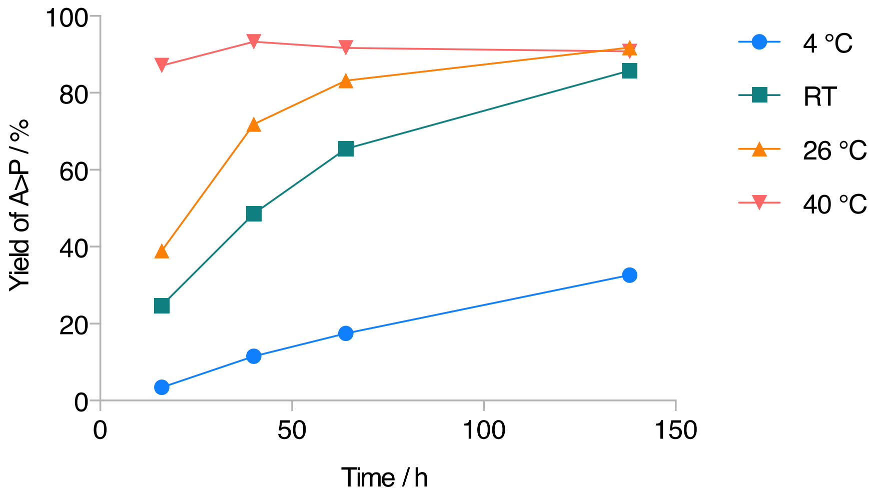

Fig. S9. The yield of A>P following reaction of A3'P with GlyGly and cyanamide-Cu^{2+}, at different temperatures. Standard reaction conditions: A3'P (12.5 mM), cyanamide (100 mM) and $CuCl_2$ (25 mM and GlyGly (50 mM), reaction times and temperatures as indicated.

Table S8. Product distribution (%) for N3'P, N>P and N2'P and overall N>P yield %, under the optimal conditions.

pH value	Nucleotide	N>P	N2'P	N3'P	Overall N>P
pH 4[a]	A3'P	89.6 %	1.3 %	9.1 %	93.1 %
	A2'3'P[b]	80.5 %	3.7 %	15.8 %	n.c.[c]
	C3'P	82.4 %	6.0 %	11.6 %	99.2 %
	G2'3'P[d]	85.7 %	3.0 %	11.1 %	n.c.[c]
pH 5.5[e]	A3'P	83.5 %	1.5 %	15.0 %	87.7 %
	A2'3'P[b]	61.3 %	4.8 %	34.0 %	n.c.[c]
	C3'P	72.4 %	8.0 %	19.6 %	94.4 %
	G2'3'P[d]	63.4 %	7.4 %	29.2 %	n.c.[c]

a. Standard reaction conditions: N3'P (12.5 mM), $CuCl_2$ (25 mM), cyanamide (100 mM), pH 4 and GlyGly (12.5 mM), 40 °C for 20 hours;
b. Starting materials contain A2'P (23 %) and A3'P (77 %);
c. Not calculable;
d. Starting materials contain G2'P (36 %) and G3'P (64 %);
e. Standard reaction conditions: N3'P (12.5 mM), $CuCl_2$ (25 mM), cyanamide (100 mM), pH 5.5 and GlyGly (50 mM), 40 °C for 20 hours.

References

S1. S. Stoll and A. Schweiger, *J. Magn. Reson.*, 2006, **178**, 42.

S2. A. R. Manyak, C. B. Murphy and A. E. Martell, *Arch. Biochem. Biophys.*, 1955, **59**, 373.
